# Assisting nurses with evidence-based practice: A case for the Knowledge-to-Action Framework

**DOI:** 10.4102/hsag.v27i0.2118

**Published:** 2022-11-02

**Authors:** Wilma ten Ham-Baloyi

**Affiliations:** 1 Faculty of Health Sciences, Nelson Mandela University, Gqeberha, South Africa

Globally, nurses are expected to base their practices on rigorous evidence to improve individual patients’ health outcomes and the overall quality of health care. Therefore, the use, uptake and implementation of best practices, such as evidence-based health practices, methods, interventions, procedures or techniques are increasingly encouraged in an attempt to provide the best care possible in an environment that has become progressively more complex (Lehane et al 2019). Evidence-based practice (EBP) requires nurses to incorporate the best research with clinical proficiency and patient values to achieve optimal health outcomes (Lehane et al. 2019; Winters & Echeverri 2012). However, it takes on average 17 years for best practices to be implemented in clinical practice (Green et al. 2009), which can be referred to as the so-called ‘knowledge-practice gap’. Although nursing students are introduced to the concept of EBP at an early stage during their nursing education, and there is an expectation for nurses to use, uptake and implement best practices, this often does not happen because of various reported barriers, including lack of time, staff shortages, heavy patient caseloads, limited knowledge of EBP and negative beliefs towards EBP as well as limited academic skills, which seems especially to be the case for novice nurses (Ferguson & Day 2007; Mallion & Brooke 2016).

**FIGURE 1:**
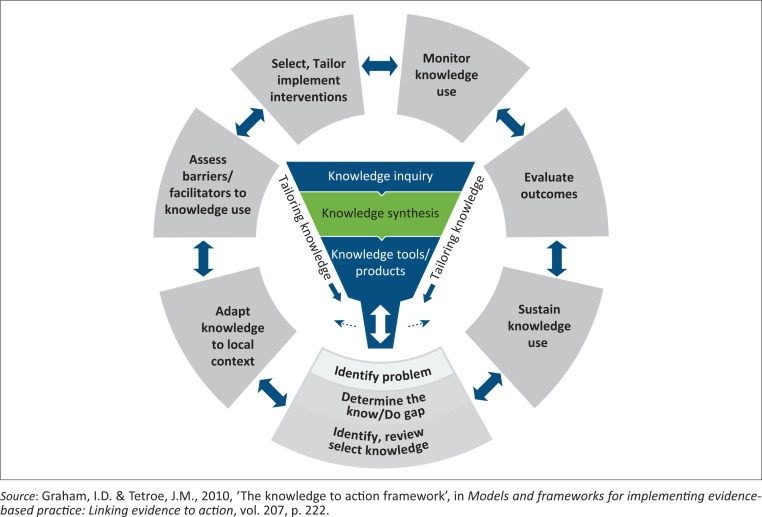
The Knowledge-to-Action framework.

Knowledge translation is used to close the knowledge-practice gap and can be defined as translating clinical science, knowledge or evidence, which aims to enhance health outcomes (Grimshaw et al. 2012; Steinskog et al. 2021). The Knowledge-to-Action (KTA) Framework was developed based on consensus of 31 planned-action theories (the action cycle) as well as the knowledge creation component to offer a holistic view of knowledge translation (Graham & Tetroe 2010). The KTA framework consists of two main components: *Knowledge Creation* and the *Action Cycle*. The *Knowledge Creation* process is divided into three phases: (1) knowledge inquiry, (2) knowledge synthesis and (3) knowledge tools and products, whereas the *Action Cycle* focuses on application of knowledge in the practice setting, using seven phases, namely: (1) identifying problem or gap that needs attention and identify, review and select the knowledge (evidence) that can solve that problem or gap; (2) adapting or tailoring the knowledge obtained to the local context; (3) assessing barriers and facilitators to knowledge use; (4) selecting, tailoring and implementing the tailored interventions; (5) monitoring the knowledge use after implementation of the interventions; (6) evaluating outcomes on the interventions’ target group and (7) sustaining the knowledge use (Graham & Tetroe 2010) (see Figure 1).

**FIGURE 2:**
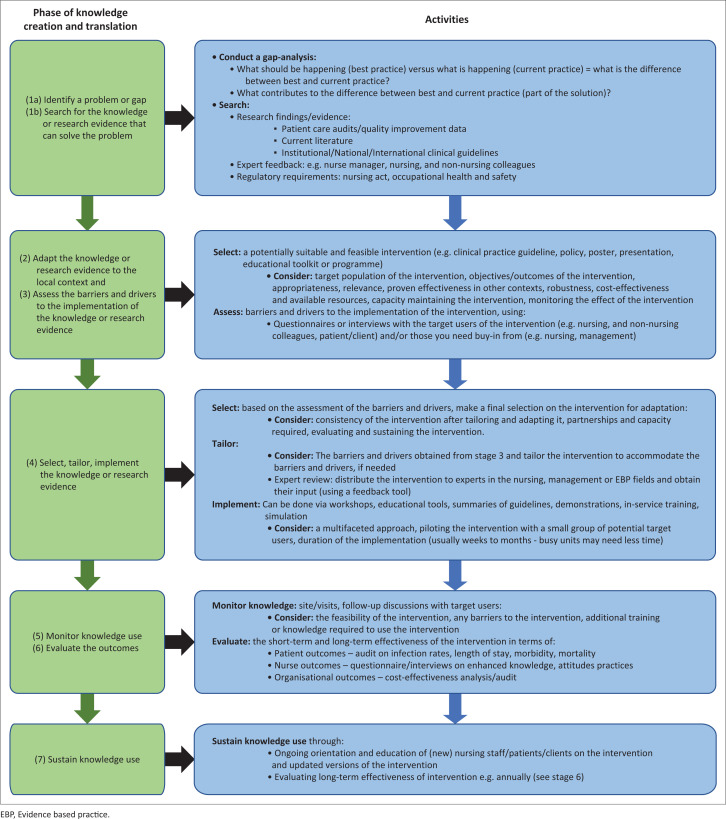
Utilisation of the knowledge-translation-action framework.

Figure 2 illustrates how the KTA framework can be used by (novice) nurses for the uptake, use and implementation of EBPs in clinical practice.

Some side notes need to be made to the proposed activities in relation to the KTA framework in Figure 2. The proposed activities are not fully comprehensive and may be adapted, depending on the knowledge translation context. Further, the distinction between adaptation of the knowledge (stage 2) and selecting the knowledge (stage 4) is not always clear or may seem repetitive; however, considering adaptability from the earliest stages of knowledge translation could help ensure that interventions are more resilient to changes in the context. Additionally, stakeholder involvement to create a sense of ownership, leadership, a feasible and contextually appropriate plan or strategy on how to implement the intervention, as well as buy-in as overarching principles should be central to all stages of knowledge translation (Moore et al. 2021). It is further recommended that novice nurses should be mentored and trained by, for example, senior nurses or academics on EBP, and active partnership and collaboration between (novice) nurses and academics must be promoted, especially as limited knowledge of EBP and limited academic skills were reported barriers towards the uptake, use and implementation of EBPs (Mallion & Brooke 2016). Nonetheless, it is expected that the various proposed activities to create and translate knowledge according to the KTA framework could be used by (novice) nurses to promote the uptake, use and implementation of EBPs in clinical settings, thereby reducing the knowledge-practice gap.
